# Antitumor Effects of Paeoniflorin on Hippo Signaling Pathway in Gastric Cancer Cells

**DOI:** 10.1155/2021/4724938

**Published:** 2021-01-19

**Authors:** Kai Niu, Yanling Liu, Zijun Zhou, Xuefeng Wu, Huaiwu Wang, Jingzhe Yan

**Affiliations:** ^1^Department of Thoracic Oncology, Jilin Province Cancer Hospital, Changchun, Jilin 130012, China; ^2^Department of Internal Medicine, Jilin Province Cancer Hospital, Changchun, Jilin 130012, China; ^3^Department of Breast Surgery, Jilin Province Cancer Hospital, Changchun, Jilin 130012, China; ^4^Department of Clinical Laboratory, Jilin Province Cancer Hospital, Changchun, Jilin 130012, China; ^5^Department of Anesthesiology, Jilin Province Cancer Hospital, Changchun, Jilin 130012, China; ^6^Department of Abdominal Oncosurgery, Jilin Province Cancer Hospital, Changchun, Jilin 130012, China

## Abstract

**Background:**

Paeoniflorin has been reported to exert antitumor effects on human cancers. However, the role of paeoniflorin in gastric cancer and the underlying molecular mechanism are unelucidated. Therefore, we determined whether paeoniflorin could exhibit anticancer activity in gastric cancer cells.

**Methods:**

MTT was used to measure the viability of cells after paeoniflorin treatment. FACS was performed to examine cell apoptosis. Wound healing and transwell invasion assays were conducted to examine cell migratory and invasive activities. Western blotting was used to explore the mechanism by which paeoniflorin exerted tumor suppressive effects.

**Results:**

We found that paeoniflorin suppressed cell growth, enhanced apoptosis, and reduced cell invasion. Notably, we showed that paeoniflorin inhibited the expression of TAZ in gastric cancer cells. The overexpression of TAZ abrogated the antitumor activity of paeoniflorin in gastric cancer cells. In contrast, the downregulation of TAZ promoted the tumor suppressive effects of paeoniflorin treatment.

**Conclusion:**

Hence, targeting TAZ with paeoniflorin could be a novel approach for the treatment of human gastric cancer.

## 1. Introduction

Gastric cancer is one of the most common malignant cancers worldwide [1]. Globally, 1,033,701 new cases of gastric cancer have been reported, and gastric cancer is the fifth most frequently diagnosed cancer. There have been an estimated 782,685 deaths due to this disease, and it is the third leading cause of cancer-related mortality [1]. There are 27,510 new gastric cancer cases and an estimated 11,140 gastric cancer-related deaths in the United States [2]. One risk factor for stomach cancer is *Helicobacter pylori* infection, which leads to most cases of gastric cancer. In addition, salty and preserved foods, low fruit intake, alcohol consumption, and smoking also contribute to gastric cancer [2]. Although multidisciplinary treatments, including surgery, chemotherapy, and immunotherapy, have been used, the five-year survival rate of gastric cancer patients is still approximately 20%–40% [3]. Therefore, it is imperative to discover new therapeutic agents to treat this deadly disease.

Paeoniflorin is obtained from the paeony root and has multiple functions, such as regulation of immunoreactions, prevention of convulsion, and protection against hypotensive syndrome [4]. Paeoniflorin has also been shown to have anticancer activities in a wide range of human malignancies [4]. For instance, paeoniflorin exerts its antiproliferative effects via regulation of cell cycle arrest and Fas/Fas ligand-dependent apoptosis in non-small cell lung cancer (NSCLC) cells [5]. Paeoniflorin was found to inhibit the expression of Bcl-2 and promote the expression of Bax and caspase-3, leading to the induction of apoptosis in cervical cancer cells [6]. Moreover, paeoniflorin suppressed growth of colorectal carcinoma cells via induction of cell cycle arrest and activation of p53, caspase-3, and caspase-9 [7]. Similarly, paeoniflorin stimulated apoptosis in hepatocellular carcinoma (HCC) cells via inhibition of prostaglandin E receptor EP2, elevation of the Bax-to-Bcl-2 ratio, and activation of caspase-3 [7]. Consistent with these findings, paeoniflorin inhibited the expression of matrix metalloproteinase (MMP-9) and extracellular signal-regulated kinase (ERK) and promoted the expression of E-cadherin in HCC cells, leading to suppression of cell migration and invasion [6]. These findings clearly suggest that paeoniflorin has antitumor activity in human cancers.

The anticancer activity of paeoniflorin in gastric cancer and the underlying mechanism have not been fully investigated. Therefore, in this study, we aimed to determine whether paeoniflorin could regulate cell viability, apoptosis, and cell cycle progression in gastric cancer. Moreover, we investigated whether paeoniflorin could control cell migration and invasion in gastric cancer. The Hippo pathway is known to play a vital role in the downregulation of tumorigenesis and progression of gastric cancer [8–10]. Hippo pathway is dysregulated and contributes to gastric oncogenesis and metastasis [10]. Yes-associated protein 1 (YAP1) and TAZ, two key factors in the Hippo pathway, exerted oncogenic function via cross-talking with Notch, TGF-*β*, Wnt/*β*-catenin in gastric cancer [11]. Therefore, we defined the molecular mechanism by which paeoniflorin exerts tumor suppressive effects via regulation of the Hippo signaling pathway in gastric cancer. Our findings provide evidence that paeoniflorin could be a potential agent to treat patients with gastric cancer.

## 2. Materials and Methods

### 2.1. Reagents

MTT (3-(4,5-dimethyl-2-thiazolyl)-2,5-diphenyl-2-H-tetrazolium bromide) was purchased from Sigma-Aldrich (St. Louis, MO, USA). Paeoniflorin was obtained from the Huanyu Biotechnology Development Company (Beijing, China). A transwell chamber assay kit was purchased from BD Biosciences. An annexin apoptosis assay kit was purchased from Beyotime Biotechnology (Shanghai, China). Anti-TAZ, anti-*β*-catenin, and anti-tubulin antibodies were purchased from Cell Signaling Technology (Danvers, MA, USA).

### 2.2. Cell Culture

Human gastric cancer MGC803 and SGC7901 cells were cultured in DMEM (Dulbecco's modified Eagle's medium) supplemented with 10% fetal bovine serum (FBS), 100 *μ*g/ml streptomycin, and 100 U/ml penicillin and incubated in a 5% CO_2_ atmosphere at 37°C.

### 2.3. MTT Assay

After MGC803 and SGC7901 gastric cancer cells were incubated in 96-well plates overnight, the cells were exposed to various concentrations of paeoniflorin for 48 h and 72h. Cell viability was measured by MTT assay, as previously described [12].

### 2.4. Cell Apoptosis Measurement

MGC803 and SGC7901 gastric cancer cells were incubated in 6-well plates overnight. Then, the cells were exposed to different concentrations of paeoniflorin for 72 h. Apoptotic cell death was measured using the annexin V-FITC/PI approach, as previously described [12].

### 2.5. Cell Cycle Analysis

MGC803 and SGC7901 gastric cancer cells were cultured and treated with different doses of paeoniflorin for 72 h. The cell cycle was measured using the propidium iodide dye method, as previously described [13].

### 2.6. Wound Healing Assay

MGC803 and SGC7901 gastric cancer cells were cultured in 6-well plates and grown to more than 90% confluence. A wound was created in the MGC803 cells using a sterile pipette tip. Then, the cells were treated with different doses of paeoniflorin for 20 h. Photographic images were captured using a microscope to show cell migration at the lesion site.

### 2.7. Transwell Chamber Invasion Assay

The treated cells were incubated in the top chamber of inserts coated with Matrigel in a 24-well plate. Complete medium was added to the bottom chamber, while serum-free medium was added to the top chamber. After the cells were cultured for 20 h, cotton buds were used to remove the cells from the top chamber. The cells in the bottom chambers were stained with calcein AM at 37°C for 1 h. The invasive cells were imaged using a fluorescence microscope.

### 2.8. Western Blotting Analysis

The treated cells were lysed in RIPA buffer. The proteins were loaded on SDS-PAGE gels and subsequently transferred to PVDF membranes. Protein expression was measured by western blotting, as previously described [13].

### 2.9. Transfection

MGC803 and SGC7901 cells were incubated in 6-well plates overnight. Plasmids containing TAZ siRNA or TAZ cDNA were transfected into MGC803 and SGC7901 cells with Lipofectamine 2000, as previously described [12].

### 2.10. Statistical Analysis

GraphPad Prism 5.0 (Graph Pad Software, La Jolla, CA) was used for statistical analysis. The results are presented as the mean values ± standard deviation (SD). ANOVA was used followed by Tukey's post-hoc test for comparisons among groups. *p* < 0.05 was considered statistically significant.

## 3. Results

### 3.1. Paeoniflorin Suppresses the Viability of Gastric Cancer Cells

**Figure 1:**
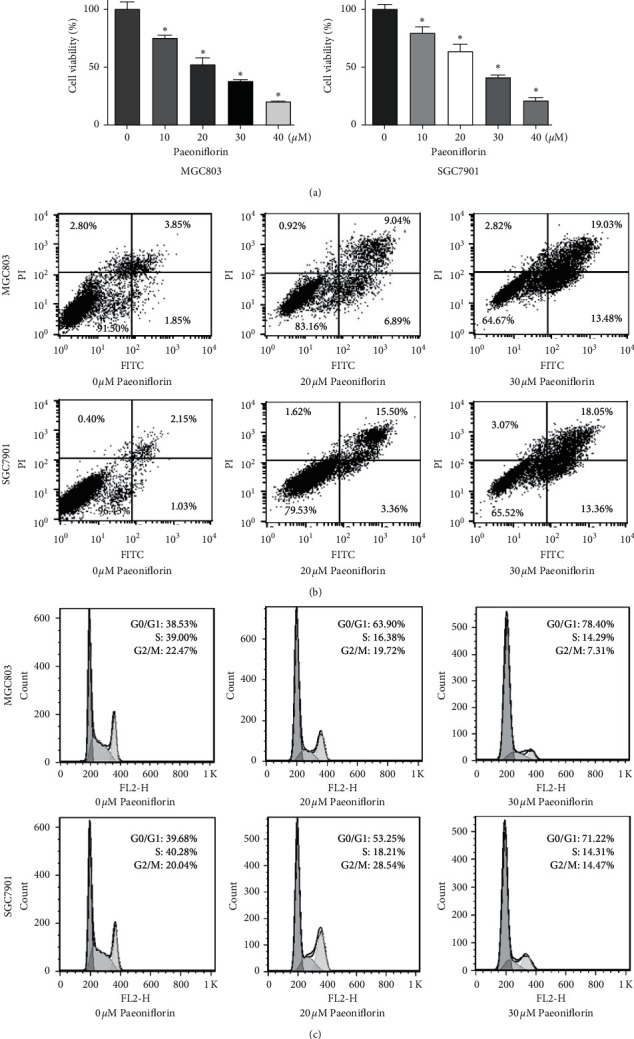
Paeoniflorin suppresses cell viability and induces apoptosis and cell cycle arrest. (a) Viability of MGC803 and SGC7901 gastric cancer cells treated with different doses of paeoniflorin for 72 h was measured by MTT. *∗p* < 0.05 vs. control groups. The results are presented as the mean ± SD. (b) Apoptosis of MGC803 and SGC7901 cells treated with paeoniflorin for 72 h was measured by flow cytometry. (c) Cell cycle analysis was measured by flow cytometry in MGC803 and SGC7901 cells after paeoniflorin exposures for 72 h.

Paeoniflorin has been shown to play an antiproliferation role in a range of human cancer cells [4]. To investigate whether paeoniflorin could suppress the viability of gastric cancer cells, MGC803 gastric cancer cells were exposed to different concentrations of paeoniflorin for 72 h. Then, an MTT  assay was performed to measure the viability of MGC803 gastric cancer cells after paeoniflorin treatment. Our MTT results showed that paeoniflorin suppressed the viability of MGC803 and SGC7901 cells in a dose-dependent manner (Figure 1(a)). Briefly, exposure to 20 *μ*M and 30 *μ*M paeoniflorin for 72 h led to 50% and 70% inhibition, respectively, of the viability of MGC803 cells (Figure 1(a)). Similar results were found in SGC7901 cells (Figure 1(a)). Thus, we selected 20 *μ*M and 30 *μ*M paeoniflorin for our subsequent studies. Our data suggest that paeoniflorin reduced cell viability in gastric cancer.

### 3.2. Paeoniflorin Induces the Apoptosis of Gastric Cancer Cells

It has been accepted that the induction of apoptosis contributes to the inhibition of cell viability in human cancer. Paeoniflorin is known to induce tumor cell apoptosis in numerous cancers [4]. Therefore, we determined whether paeoniflorin could regulate the apoptosis of gastric cancer cells. An annexin V-FITC/PI assay was utilized to assess the apoptotic death of gastric cancer cells after paeoniflorin treatment. We observed that paeoniflorin increased the degree of MGC803 gastric cancer cell apoptosis from 5.7% in the control group to 15.93% in the 20 *μ*M paeoniflorin treatment group and 32.51% in the 30 *μ*M paeoniflorin treatment group after 72 h (Figure 1(b)). Paeoniflorin also stimulated apoptosis in SGC7901 cells (Figure 1(b)). These results demonstrated that paeoniflorin stimulated the apoptosis of gastric cancer cells.

### 3.3. Paeoniflorin Triggers Cell Cycle Arrest in Gastric Cancer Cells

Cell cycle arrest often occurs after paeoniflorin treatment in a variety of human cancers [4]. Thus, cell cycle analysis in MGC803 gastric cancer cells treated with paeoniflorin was performed by using PI staining and flow cytometry. We found that paeoniflorin triggered cell cycle arrest at the *G*1/*G*1 phase in the MGC803 cells (Figure 1(c)). In detail, 20 *μ*M and 30 *μ*M paeoniflorin treatment increased the percentage of gastric cancer cells arrested at the *G*0/*G*1 phase from 38.53% in the control group to 63.9% in the 20 *μ*M paeoniflorin group and 78.4% in the 30 *μ*M paeoniflorin group (Figure 1(c)). Clearly, paeoniflorin also induced the cell cycle arrest of SGC7901 gastric cancer cells (Figure 1(c)).

### 3.4. Paeoniflorin Inhibits the Migration  and  Invasion of Gastric Cancer Cells

**Figure 2:**
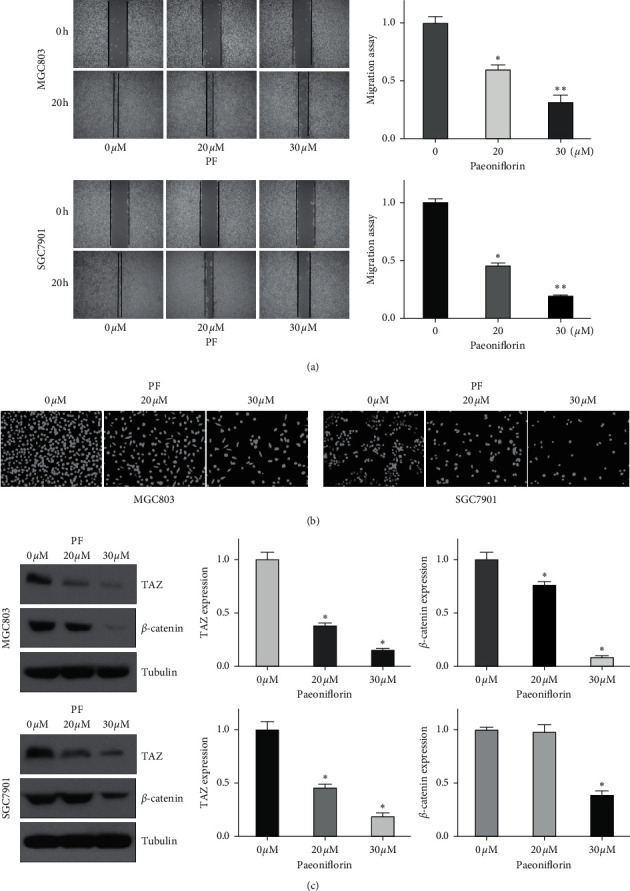
Paeoniflorin inhibits the migration and invasion of gastric cancer cells. (a) Left panel: migratory activity of MGC803 and SGC7901 cells after paeoniflorin treatments was detected by wound healing assay. Right panel: quantitative results were shown for left panel. *∗p* < 0.05, *∗∗p* < 0.01 vs. control groups. The results are presented as the mean ± SD. (b) Invasion of MGC803 and SGC7901 cells treated with paeoniflorin was detected by transwell chambers assay. Right panel: quantitative results were shown for left panel. (c) The expression of TAZ and *β*-catenin in MGC803 and SGC7901 cells after paeoniflorin treatments was measured by western blotting analysis. Right panel: quantitative results were shown for left panel.

A line of evidence has suggested that paeoniflorin inhibits cell migration and invasion in human cancers [4, 14, 15]. To determine whether paeoniflorin regulated the cell migratory and invasive activities of gastric cancer cells, we utilized a wound healing assay to measure the migration of MGC803 and SGC7901 cells. Our data showed that paeoniflorin treatment inhibited wound healing in the gastric cancer cells, suggesting that paeoniflorin suppressed cell migration (Figure 2(a)). Moreover, a Transwell chamber assay was utilized to detect the invasiveness of gastric cancer cells after paeoniflorin treatment. We found that paeoniflorin delayed the invasion of gastric cancer cells (Figure 2(b)). Altogether, these results showed that paeoniflorin inhibited the motility of gastric cancer cells.

### 3.5. Paeoniflorin Downregulates the Expression of TAZ

It has been reported that transcriptional coactivator with PDZ-binding motif (TAZ) is an important factor in Hippo signaling pathway, and that it plays a vital role in gastric cancer development and progression [16]. Therefore, we tested whether paeoniflorin could regulate the expression of TAZ in gastric cancer cells. Our Western blotting analysis results showed that paeoniflorin downregulated the TAZ expression levels in the MGC803 and SGC7901 cells (Figure 2(c)). Moreover, *β*-catenin, a downstream target of TAZ, was inhibited by paeoniflorin treatment in gastric cancer cells (Figure 2(c)). Together, these data suggested that paeoniflorin suppressed the expression of TAZ in gastric cancer cells.

### 3.6. TAZ Overexpression Abrogates Paeoniflorin-Mediated Cell Viability Inhibition and Apoptosis

**Figure 3:**
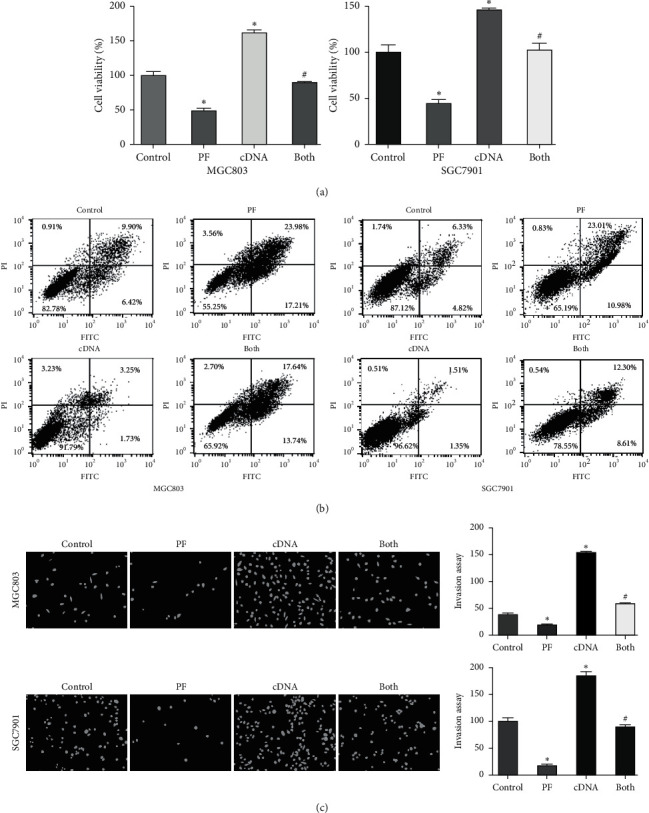
Overexpression of TAZ reverses the paeoniflorin-mediated cell growth inhibition. (a) Viability of MGC803 and SG7901 cells after TAZ overexpression and paeoniflorin treatment was determined by MTT assay. The results are presented as the mean ± SD. *∗p* < 0.05 vs. control groups; ^#^*p* < 0.05 vs. PF alone or cDNA transfection alone. PF: 20 *μ*M paeoniflorin; cDNA: TAZ cDNA; both: PF exposure and TAZ cDNA transfection. (b) Apoptosis of gastric cancer cells after TAZ overexpression and paeoniflorin treatment was measured by flow cytometry. (c) Left panel: invasion of gastric cancer cells overexpressing TAZ and treated with paeoniflorin was assessed by transwell chambers assay. Right panel: quantitative results were represented for left panel.

Next, we performed a rescue experiment to validate whether TAZ is involved in the anti-tumor activity of paeoniflorin in gastric cancer cells. Gastric cancer cells were transfected with TAZ cDNA to upregulate the expression of TAZ and then treated with paeoniflorin for 72 h. The results from the MTT assay revealed that the overexpression of TAZ enhanced the viability of the MGC803 and SGC7901 cells (Figure 3(a)). Importantly, the overexpression of TAZ abrogated the paeoniflorin-induced inhibition of viability in the gastric cancer cells (Figure 3(a)). Moreover, we found that the upregulation of TAZ inhibited the apoptosis of gastric cancer cells (Figure 3(b)). The overexpression of TAZ rescued the paeoniflorin-induced apoptosis of gastric cancer cells (Figure 3(b)). These data suggest that TAZ is critically involved in the antitumor activity of paeoniflorin in gastric cancer.

### 3.7. TAZ Overexpression Reduces the Effects of Paeoniflorin on Migration and Invasion

**Figure 4:**
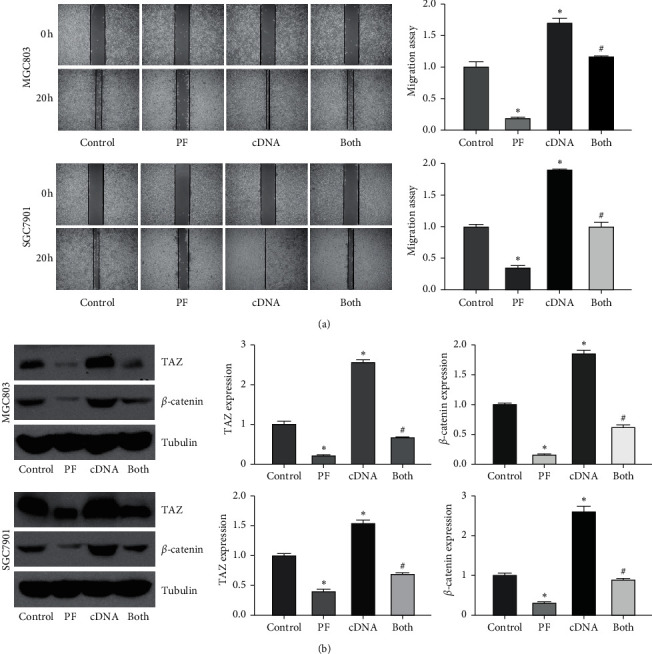
Overexpression of TAZ reverses paeoniflorin-mediated inhibition of migration and invasion. (a) Left panel: migration of gastric cancer cells overexpressing TAZ and treated with paeoniflorin was detested by wound healing assay. Right panel: quantitative results were represented for left panel. The results are presented as the mean ± SD. PF: 20 *μ*M paeoniflorin; cDNA: TAZ cDNA; both: PF exposure and TAZ cDNA transfection. (b) The expression of TAZ and *β*-catenin in gastric cancer cells overexpressing TAZ and treated with paeoniflorin was measured by western blotting analysis. Right panel: quantitative results were shown for left panel.

We examined whether the inhibition of migration and invasion by paeoniflorin is due to the suppression of TAZ in gastric cancer cells. Our transwell chamber assay data showed that the overexpression of TAZ promoted the invasiveness of gastric cancer cells (Figure 3(c)). Increased expression of TAZ by cDNA transfection abrogated the suppression of gastric cancer cell invasion by paeoniflorin (Figure 3(c)). The migratory activity of MGC803 cells was detected after TAZ cDNA transfection and paeoniflorin treatment for 72 h. Our wound healing assay showed that the overexpression of TAZ increased the migration of gastric cancer cells (Figure 4(a)). The upregulation of TAZ abolished the paeoniflorin-triggered inhibition of gastric cancer cell migration (Figure 4(a)). Furthermore, our western blotting results demonstrated that the overexpression of TAZ increased the expression of *β*-catenin, which was reduced by paeoniflorin treatment in gastric cancer cells (Figure 4(b)). In summary, TAZ plays an essential role in the paeoniflorin-mediated inhibition of the motility of gastric cancer cells.

### 3.8. TAZ Downregulation Enhances the Tumor Suppressive Activity of Paeoniflorin

**Figure 5:**
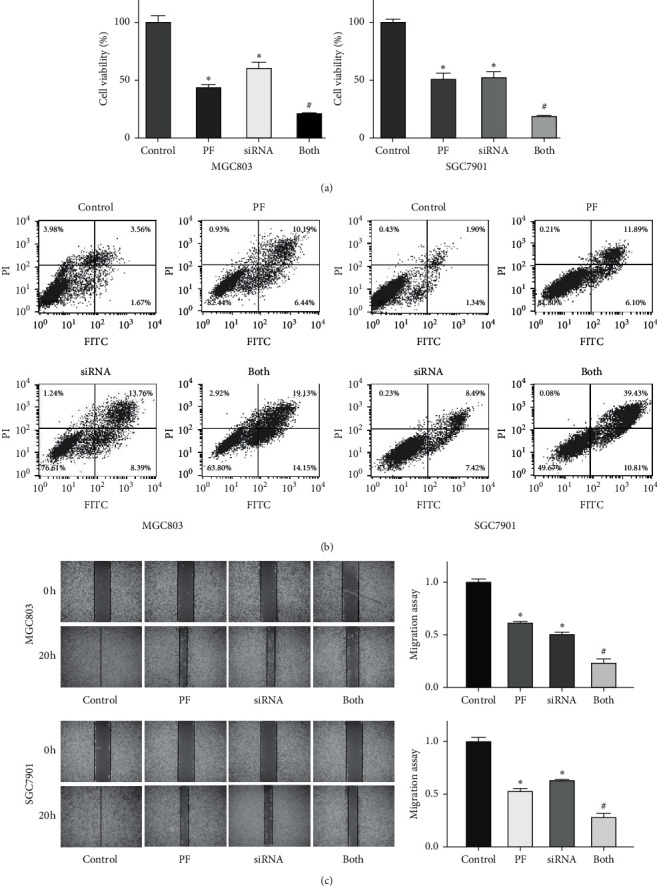
Downregulation of TAZ promotes the paeoniflorin-mediated cell growth inhibition. (a) Viability of gastric cancer cells with downregulated TAZ and treated with paeoniflorin was determined by MTT  assay. The results are presented as the mean ± SD. *∗p* < 0.05 vs. control groups; ^#^*p* < 0.05 vs. PF alone or siRNA transfection alone. PF: 20 *μ*M paeoniflorin; siRNA: TAZ siRNA; both: PF exposure and TAZ siRNA transfection. (b) Apoptosis of cells with downregulated TAZ and treated with paeoniflorin was measured by flow cytometry. (c) Left panel: migration of gastric cancer cells with downregulated TAZ and treated with paeoniflorin was detested by wound healing assay. Right panel: quantitative results were represented for left panel.

**Figure 6:**
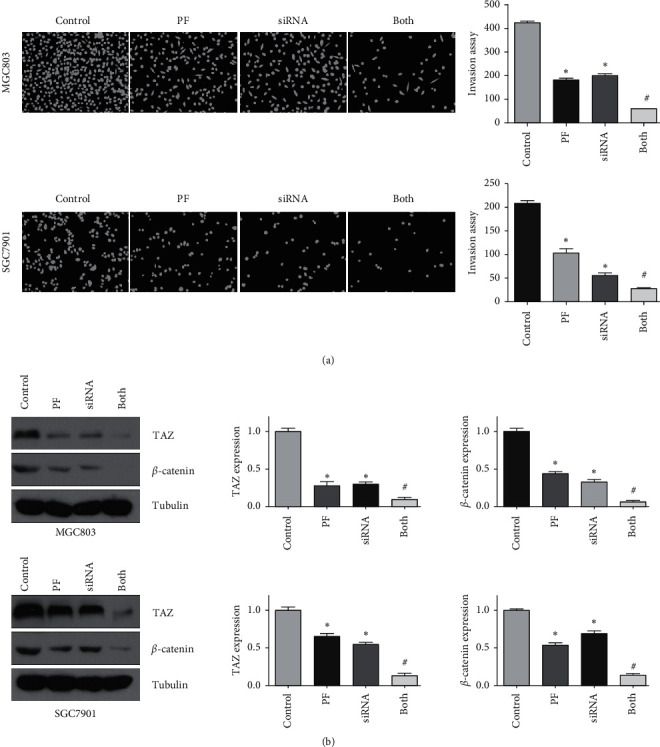
Downregulation of TAZ promotes the paeoniflorin-mediated tumor suppressive activity. (a) Left panel: invasion of gastric cancer cells with downregulated TAZ and treated with paeoniflorin was assessed by transwell chambers assay. Right panel: quantitative results were represented for left panel. The results are presented as the mean ± SD. ^*∗*^*p* < 0.05 vs. control groups; ^#^*p* < 0.05 vs. PF alone or siRNA transfection alone. PF: 20 *μ*M paeoniflorin; siRNA: TAZ siRNA; both: PF exposure and TAZ siRNA transfection. (b) The expression of TAZ and *β*-catenin in gastric cancer cells with downregulated TAZ and treated with paeoniflorin was measured by western blotting analysis. Right panel: quantitative results were shown for left panel.

To thoroughly confirm the role of TAZ in the anticancer activity of paeoniflorin, TAZ siRNA was transfected into gastric cancer cells after paeoniflorin treatment. The MTT approach was used to measure cell viability, and our data showed that the downregulation of TAZ decreased the viability of gastric cancer cells (Figure 5(a)). Moreover, the downregulation of TAZ enhanced the paeoniflorin-induced inhibition of the viability of gastric cancer cells (Figure 5(b)). Furthermore, TAZ siRNA transfection induced cell apoptosis and promoted paeoniflorin-induced apoptosis of gastric cancer cells (Figure 5(b)). In addition, the downregulation of TAZ retarded the migration and invasion of gastric cancer cells (Figures 5(c) and 6(a)). Strikingly, the downregulation of TAZ promoted the suppression of motility in gastric cancer cells by paeoniflorin (Figures 5(c) and 6(a)). Our western blotting data indicated that the downregulation of TAZ enhanced the suppression of *β*-catenin expression by paeoniflorin in gastric cancer (Figure 6(b)). Taken together, these data showed that paeoniflorin exerted its anticancer effects in part via inhibition of TAZ in gastric cancer.

## 4. Discussion

Accumulating evidence has revealed that paeoniflorin decreases antiproliferation and increases apoptosis in human cancer cells [4]. One study showed that paeoniflorin triggered cell growth inhibition and apoptosis via the enhancement of STAT3 degradation in glioma cells [17]. Paeoniflorin suppressed cell proliferation by inhibiting Notch-1 signaling pathway in breast cancer cells [15]. In addition, paeoniflorin enhanced cell apoptosis in pancreatic cancer cells via the inhibition of MMP-9 and ERK signaling pathways [18]. Yang et al. found that paeoniflorin reduced cell growth via the inactivation of signal transducer and activator of transcription 3 (STAT3) in bladder carcinoma [19]. Moreover, paeoniflorin was reported to repress cell viability and stimulate apoptosis in MGC803 cells via the upregulation of miR-124 and inactivation of phosphatidylinositol 3-kinase (PI3K)/Akt and STAT3 signaling [20]. In the present study, our results demonstrate that paeoniflorin inhibited cell viability and triggered apoptosis in gastric cancer cells. This finding suggests that paeoniflorin could be a promising agent to inhibit tumor cell growth in gastric cancer.

A line of evidence has demonstrated that paeoniflorin could retard cell motility in various types of human cancers [4, 14, 15]. A study reported that paeoniflorin suppressed the invasion of breast cancer cells by targeting Notch-1 pathway [15]. Moreover, paeoniflorin blocked cell migration and invasion through the inhibition of transforming growth factor beta-mediated epithelial-mesenchymal transition in glioblastoma cells [14]. Furthermore, paeoniflorin inhibited cell migration and invasion in gastric cancer associated fibroblasts through the upregulation of miR-149 expression and inactivation of interleukin 6 (IL-6)-STAT3-matrix metalloproteinase (MMPs), indicating that paeoniflorin could be a new agent that targets the cancer microenvironment [14]. The effects of paeoniflorin on the migration and invasiveness of gastric cancer cells have not been reported. Herein, our study showed that paeoniflorin retarded the migratory and invasive activities of gastric cancer cells, indicating that paeoniflorin might be a potential compound to inhibit tumor metastasis in gastric cancer.

Several studies have demonstrated that paeoniflorin exerts tumor suppressive effects via different molecular mechanisms in gastric cancer [7, 14, 21, 22]. For example, paeoniflorin suppressed the activity of NF-kappaB in SGC7901 gastric carcinoma cells via the prevention of IkappaB-alpha phosphorylation [7]. Furthermore, paeoniflorin enhanced the sensitivity of gastric carcinoma cells to 5-fluorouracil-mediated apoptosis [7]. A subsequent study showed that paeoniflorin inhibited the expression of multidrug resistance (MDR) by reducing NF-kappaB activation and subsequently inhibiting its downstream targets, such as MDR1, Bcl-XL, and Bcl-2, in SGC7901 cells [21]. In the current study, we showed that paeoniflorin inhibited the expression of TAZ in gastric cancer cells. It is accepted that TAZ is a core effector in Hippo signaling pathway. TAZ overexpression was observed in gastric cancer tissues and was associated with lymphatic metastasis and tumor TNM stage in gastric cancer [8]. Moreover, TAZ overexpression has also been observed in gastric signet ring cell carcinoma [23]. Furthermore, YAP/TAZ was characterized to act as an oncogenic initiator and driver of gastric carcinogenesis via an increase in MYC expression [24]. Notably, TAZ overexpression is involved in cisplatin resistance in gastric cancer cells via the induction of EMT [25]. These reports indicate that TAZ is an important factor in gastric tumorigenesis and progression. It is necessary to mention that the current study had several limitations. First, a limitation is the lack of comparison with an approved efficacious drug for the treatment of gastric cancer in order to investigate the antitumor effect of paeoniflorin. Second, as the downregulation of TAZ induced by paeoniflorin was evaluated only in terms of protein level in the gastric cancer cells by western blotting analysis, a gene expression analysis should be performed. Third, other downstream factors of the Hippo signaling pathway should be evaluated to better understand the antitumor activity of paeoniflorin in gastric cancer. Four, an in vivo study evaluating not only the effectiveness of paeoniflorin but also its toxicity and tolerability is required before suggesting a potential use for the treatment of gastric cancer patients. Therefore, in-depth investigation is necessary to determine the functions of paeoniflorin and its underlying mechanisms in gastric cancer.

## 5. Conclusion

Because paeoniflorin inhibited the expression of TAZ in gastric cancer, paeoniflorin might be considered a potential inhibitor of TAZ for the treatment of gastric cancer patients in the future.

## Data Availability

The data used to support the findings of this study are available from the corresponding author upon request.
